# A Review of Biosensors for Detecting Tumor Markers in Breast Cancer

**DOI:** 10.3390/life12030342

**Published:** 2022-02-25

**Authors:** Rui Hong, Hongyu Sun, Dujuan Li, Weihuang Yang, Kai Fan, Chaoran Liu, Linxi Dong, Gaofeng Wang

**Affiliations:** 1 Ministry of Education Engineering Research Center of Smart Microsensors and Microsystems, Hangzhou Dianzi University, Hangzhou 310018, China; hongrui@hdu.edu.cn (R.H.); sunhongyu@hdu.edu.cn (H.S.); yangwh@hdu.edu.cn (W.Y.); fankai@hdu.edu.cn (K.F.); liucr@hdu.edu.cn (C.L.); donglinxi@hdu.edu.cn (L.D.); gaofeng@hdu.edu.cn (G.W.); 2 School of Automation, Hangzhou Dianzi University, Hangzhou 310018, China; 3 School of Electronics and Information, Hangzhou Dianzi University, Hangzhou 310018, China

**Keywords:** tumor marker, breast cancer, biosensor

## Abstract

Breast cancer has the highest cancer incidence rate in women. Early screening of breast cancer can effectively improve the treatment effect of patients. However, the main diagnostic techniques available for the detection of breast cancer require the corresponding equipment, professional practitioners, and expert analysis, and the detection cost is high. Tumor markers are a kind of active substance that can indicate the existence and growth of the tumor. The detection of tumor markers can effectively assist the diagnosis and treatment of breast cancer. The conventional detection methods of tumor markers have some shortcomings, such as insufficient sensitivity, expensive equipment, and complicated operations. Compared with these methods, biosensors have the advantages of high sensitivity, simple operation, low equipment cost, and can quantitatively detect all kinds of tumor markers. This review summarizes the biosensors (2013–2021) for the detection of breast cancer biomarkers. Firstly, the various reported tumor markers of breast cancer are introduced. Then, the development of biosensors designed for the sensitive, stable, and selective recognition of breast cancer biomarkers was systematically discussed, with special attention to the main clinical biomarkers, such as human epidermal growth factor receptor-2 (HER2) and estrogen receptor (ER). Finally, the opportunities and challenges of developing efficient biosensors in breast cancer diagnosis and treatment are discussed.

## 1. Introduction

Nowadays, cancer has become one of the main threats to human health and life [1]. Among all types of cancer, breast cancer has the highest incidence rate in women worldwide, and the incidence rate is still increasing [2]. Previous studies have suggested that early breast cancer detection with suitable treatment could reduce breast cancer death rates significantly in the long term. At present, the main diagnostic techniques available for the detection of breast cancer are mammography, breast ultrasound, and breast MRI examination [3,4]. However, these methods require the corresponding equipment, professional practitioners, and expert analysis, and the detection cost is high. As a result, these methods are difficult to generalize to the majority of people who need this screening, especially for those with early-stage breast cancer that has not yet been detected. Compared with the above methods, the detection of the tumor markers of breast cancer using a biosensor is a more efficient and less costly [5].

A biosensor is commonly defined as a self-contained small analytical device that combines biological recognition system and a physiochemical transducer for the detection of target molecules by converting the recognition signal into a detectable output signal [6,7,8,9,10]. It is able to meet the needs of this group of patients. On the one hand, breast tumor markers play an important role in the early diagnosis of breast cancer, the classification of molecular subtypes, the choice of treatment methods, and the prognosis evaluation [11,12,13]. On the other hand, biosensors offer significant advantages in terms of specificity, sensitivity, speed, and cost of detection compared to traditional tumor marker detection methods [14,15] such as chemiluminescence immunoassay [16], enzyme-linked immunosorbent assay [17], proteomics [16], molecular biology methods, liquid biopsy, etc. In the last decade, numerous biosensors have been developed that exhibit better sensitivity, selectivity, stability, and low cost [18]. According to the detection principle and detection signal, biosensors can be divided into electrochemical biosensors, optical biosensors, and other types [9,10,18,19,20]. Although most reported biosensors are still in the experimental stage, they are expected to be commercialized in the future to improve the effect and efficiency of tumor markers detection. 

In this review, the reported breast tumor biomarkers are summarized firstly. Then, the research progress of biosensors designed for sensitive, stable, and selective identification of breast cancer biomarkers is systematically reviewed based on the main breast cancer biomarkers. The detection strategies of the biosensors were focused on electrochemical, optical, and others such as quartz crystal microbalance. In addition, new strategies that can be combined with biosensors and improve their performance were introduced. Finally, the latest challenges and further opportunities for developing effective biosensors for the diagnosis and treatment of breast cancer are discussed.

## 2. Tumor Markers

**Figure 1:**
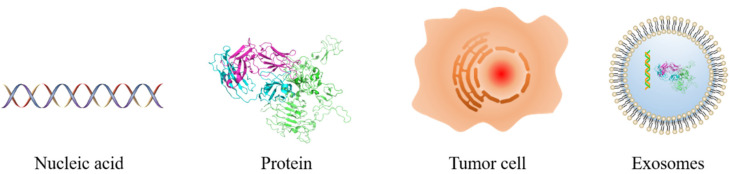
Schematic diagram of the different types of tumor markers.

Tumor markers are a class of active substances produced by the interaction between tumor tissue or host and tumor, which can indicate the existence and growth of the tumor. At any stage, the concentration of tumor markers is determined by several parameters of the tumor, such as the size, mass, expression degree, catabolic, excretion rates, tumor bloody supply, and drug resistance. As shown in Figure 1, tumor markers can be divided into nucleic acid, protein, tumor cell, and others such as the exosome.

The most commonly used breast tumor markers are estrogen receptor, progesterone receptor, and human epidermal growth factor receptor 2 [21]. In addition, the role and potential of emerging biomarkers in the diagnosis and treatment of breast cancer are also being studied and discovered.

### 2.1. Estrogen Receptor

Estrogen receptor (ER) is a protein molecule that specifically binds to estrogen in cells [22]. Estrogen receptors can be located in the cell membrane, cytoplasm, or nucleus. The classical nuclear receptor is located in the nucleus, and its protein is located in the cytoplasm temporarily after translation, so it can be detected in the cytoplasm [23]. Estrogen diffuses into the nucleus and binds to its nuclear receptor, which triggers gene regulation mechanism and regulates the transcription of downstream genes. The purpose of estrogen receptor detection is to determine whether patients are suitable for endocrine therapy, to assist in prognostication, and as a diagnostic aid in metastatic breast cancer [23,24]. Estrogen stimulates tumor growth in some breast cancer patients, and estrogen exerts its effect by binding to estrogen receptors. Therefore, the detection of patients with estrogen receptors is positive, and can determine whether the patient is suitable for endocrine therapy. Endocrine therapy for ER-positive patients can effectively inhibit tumor growth. The same treatment for ER-negative patients could not achieve the same effect [25]. There is unequivocal evidence that patients with cancers devoid of ER expression do not benefit from endocrine treatment [26].

### 2.2. Progesterone Receptor

Progesterone receptor (PR) is a hormone receptor such as ER. PR is activated by ER, and the activation of PR is the signal of ER activity [27]. The interaction between PR and chromatin will change the binding position of ER and chromatin and then lead to the change in cell gene regulation from proliferation to cell cycle arrest, apoptosis, and differentiation [23]. PR-positive patients account for about 65–70% of breast cancer patients, and PR-positive patients rarely appear ER-negative at the same time [27]. Therefore, for those patients that are strongly PR positive and ER negative, detection of ER again is needed to eliminate the possibility of a false negative [25,27]. The main purpose of PR detection is to judge the prognosis of ER-positive patients [23].

### 2.3. Human Epidermal Growth Factor Receptor 2

The human epidermal growth factor receptor 2 (HER2) gene is one of the most widely studied proto-oncogenes of breast cancer [28]. HER2 drives tumor growth by activating the MAPK and PI3K/AKT signaling pathways, which in turn enhances cell proliferation, invasion, and metastasis [23]. In the absence of systemic therapy, HER2 gene amplification or protein expression is associated with poor prognosis [29]. It was found that the HER2 level was negatively correlated with the ER and PR levels [23]. HER2-positive patients account for about 15–20% of breast cancer patients [28]. In clinical practice, targeted therapy for HER2 is adopted in HER2-positive patients and HER2 is used as a prognostic indicator [30,31]. Similar to ER, targeted therapy for HER2 has an obvious effect only on HER2-positive patients, but not on HER2-negative patients [23].

### 2.4. The Biomarker of Triple Negative Breast Cancer (TNBC)

Within the spectrum of breast cancer, TNBC is known as a type of breast cancer in which there is a lack of expression of ER, PR, and HER2 [32]. TNBC accounts for approximately 15–20% of breast cancer patients and is a more aggressive and heterogeneous subtype than other subtypes, with a poorer prognosis and less survival rates after treatment. Currently, cytotoxic chemotherapy is still the main way for the treatment of TNBC. Therefore, further classification of TNBC is needed for a more targeted and effective treatment plan [29,33]. Current research on TNBC-related biomarkers has identified a number of biomarkers that can be used to further classify patients with TNBC for molecular therapy [33,34]. In addition to this, there are also some biomarkers that indicate the prognosis and treatment status of the TNBC patients.

The signaling of angiogenesis, mediated by vascular endothelial growth factor (VEGF), is crucial in the process of growth and tumor spreading [32]. VEGF is highly expressed in around 30–60% of patients with TNBC [33], with significantly higher levels of VEGF and shorter survival times for patients with primary operable triple-negative breast cancer. Clinical trials have found that adding targeted anti-VEGF therapy to patients with TNBC improves treatment outcomes [34].

Androgen receptor (AR) is also a hormone receptor that specifically binds to androgen in cells, which modulates the transcription factors and controls gene expression [27,35]. AR can both stimulate proliferation and dedifferentiation and induce apoptosis and cell death, depending on the simultaneously activated signaling pathways [33]. The expression of AR is related to the biological behaviors of triple-negative breast cancer, and plays a role in endocrinotherapy and prognostic prediction [36].

### 2.5. Emerging Tumor Markers

In addition to the three common clinical breast cancer tumor markers mentioned above, there are many emerging tumor markers that are receiving increasing attention from researchers. These emerging tumor markers can be classified as nucleic acids, proteins, tumor cells, and others [37].

#### 2.5.1. Nucleic Acids

Nucleic acid tumor markers include BRCA1, BRCA2, microRNA, circulating tumor DNA (ctDNA), circulating cell-free DNA (ccfDNA), circulating RNA (circRNA), long noncoding RNAs(lncRNA), etc. [38]. BRCA1 and BRCA2 are the tumor-suppressor genes in breast cancer [39]. Research shows that their mutations will increase the risk of breast cancer [39,40,41]. 

MicroRNA is a kind of noncoding single-stranded RNA molecule with a length of about 22 nucleotides encoded by endogenous genes, which plays an important role in controlling gene expression [42]. MicroRNAs have emerged as key regulators of breast cancer pathogenesis, progression, and treatment response [43]. Studies have found that microRNA is related to the clinical and pathological characteristics of patients, and can target different genes and affect different pathways. The plasma microRNA-21 in breast cancer patients is about four times that of normal people; also, a significant negative correlation between its basal expression, expression levels after treatment, and time to progression in HER2-positive patients was found with the progression time [44]. 

Circulating cell-free DNA is extracellular DNA in plasma or serum and ctDNA is ccfDNA released from tumor cells into blood [45,46]. There is a significant correlation between the amount of ccfDNA and the clinical manifestations and prognosis of metastatic breast cancer patients [45]. It can serve as a tumor marker to assist the treatment of breast cancer. Primary tumor cells, circulating tumor cells, and occult and dominant metastatic tumor cells release DNA at a higher rate than normal cells, and ctDNA displays mutations characteristic of the progenitor tumor [37]. Therefore, ctDNA has the potential as a tumor marker for the prediction, diagnosis, and prognosis of breast cancer [37,46]. However, the amount of ctDNA is very low; the total amount of ctDNA may represent as low as 0.01% of the total cfDNA. It needs a more sensitive and specific detection method [37].

Different from common RNA, circRNA is a kind of double-stranded closed RNA, which is not affected by RNA exonuclease, stable expression, and is difficult to decompose. The results showed that circRNA expression was related to the proliferation, migration, invasion, and drug resistance of tumor cells; the level of hsa_circ_103110, hsa_circ_104689, hsa_circ_0058514, hsa_circ_0001982, hsa_circ_104821, hsa_circ_0001785, circKIF4A, and cirs-7 rose in the tissue of breast cancer patients, and the level of hsa_circ_406697, hsa_circ_100219, hsa_circ_006054, circTADA2As, circ_Foxo3, and circRNA _000911 declined [47,48]. LncRNA is a kind of noncoding RNA with a length over 200nt, and it is a transcription product of RNA polymerase II. LncRNA not only can be used as a marker for prediction, prognosis, and progression but also can stimulate tumor growth and increase drug resistance [49,50]. However, further research is needed to apply it to clinical diagnosis and treatment [49].

#### 2.5.2. Proteins

There are a lot of emerging protein tumor markers, such as CD24, CD44, MUC1, etc. CD24 is a glycosylphosphatidylinositol-binding glycoprotein with a large number of n- and o-type glycochains [11,51]. At present, a large number of CD24 expressions have been found in a variety of cancers [52]. It was found that CD24 is an anti-phagocytic signal, which protects cancer cells from macrophage attacks expressing Siglec-10. The blocking treatment of CD24 can effectively improve the therapeutic effect of CD24-positive tumors [51]. The expression of CD24 is also associated with the grading and staging of breast cancer [52]. CD44 is a complex transmembrane-binding glycoprotein [53,54], which can be involved in the regulation of many important signaling pathways such as tumor proliferation, invasion, metastasis, and treatment resistance, and was related to the adverse prognosis of patients [52,53].

MUC1 (CA15-3) is a transmembrane mucin glycoprotein, which is expressed in most epithelial tissues [55]. In 90% of breast cancer cases, abnormal expression and abnormal glycosylation were found in MUC1 [56]. At the same time, MUC1 is also an important marker for monitoring metastatic breast cancer [57]. In addition, the commonly used serum tumor markers, such as CEA, CA19-9, CA125, CA15-3, and TPS, play an important role in breast cancer diagnosis and treatment. In metastatic breast cancer, CEA and CA15-3 can be used to differentiate bone metastases and other metastases. CA15-3 and TPS were significantly increased in patients with liver metastasis. When the TPS level was normal and other tumor markers were increased, patients may be suspected of lung metastasis [58].

#### 2.5.3. Tumor Cells

In addition to nucleic acids and proteins, which are biomolecules, tumor cells themselves are also tumor markers. When breast cancer cells fall off from the tumor and enter into the blood system, it would be called the circulating tumor cells (CTC). CTC has the ability to form new tumor tissues. By detecting the level of CTC cells, metastatic breast cancer patients could be staged and graded to carry out targeted treatment [59]. CTC cells can also be used to evaluate the prognosis of breast cancer patients, and to determine whether patients are suitable for postoperative additional radiation therapy [37,60].

#### 2.5.4. Others

In addition to nucleic acids, proteins, and cellular tumor markers, other tumor markers, such as exosomes, are also present. Exosomes are 40–180 nm extracellular vesicles containing RNA and protein [61]. These proteins and RNA are involved in the pathogenesis of breast cancer [62]. Studies have found that tumor cells release more exosomes than normal cells, and miRNA-21 and miRNA-1246 in exosomes are upregulated in patients’ plasma [4]. Therefore, exosomes have potential and value as a tumor marker for breast cancer.

ER, PR, and HER2 are the most widely used tumor markers in the diagnosis and treatment of breast cancer. The three markers are used to classify the luminal subtypes of breast cancer in breast cancer patients, to determine the treatment of patients, and is also an important indicator for prognosis monitoring [13,27]. There are many new kinds of tumor markers. They have rich functions in the diagnosis and treatment of breast cancer. These functions include the early diagnosis of breast cancer, the choice of treatment methods, the targets of targeted therapy to cancer progression, prognosis evaluation, and drug resistance. However, whether they can be used in clinical diagnosis and treatment still needs to be further studied. At present, the current and emerging markers of breast cancer are not able to predict breast cancer effectively before the onset of clinical symptoms [39]. High specificity and high sensitivity tumor markers still need further study on breast cancer.

## 3. Biosensor

Biosensors can be divided into electrochemical biosensors, optical biosensors, and other types according to the detection principle and detection signal [6,7,8,9,10]. In recent years, researchers have developed many biosensors for detecting breast tumor markers. The research progress in electrochemical biosensors, optical biosensors, and other types for breast tumor markers is reviewed in this paper.

### 3.1. Electrochemical Biosensor

An electrochemical biosensor realizes the quantitative detection of the target by detecting the electrochemical reaction on the surface of the electrode. The signal of the electrochemical reaction is related to the target concentration [63,64,65]. The main methods of electrochemical detection are cyclic voltammetry (CV), differential pulse voltammetry (DPV), square wave voltammetry (SWV), linear sweep voltammetry (LSV), electrochemical impedance spectroscopy (EIS), field-effect biosensor (FET), and other methods [6,7,18].

#### 3.1.1. Cyclic Voltammetry

The principle of cyclic voltammetry is to apply a triangle wave voltage in the form of fast linear scanning; complete a cycle of the reduction process and oxidation process with one triangle wave scanning; and then analyze according to the current–potential curve [66].

For example, Hong et al. [67] modified ferrocenecarboxylic (Fc-COOH)-doped silica nanoparticles (SNPs) on the surface of the Au electrode to connect with glutaraldehyde that could bind with the CA15-3 antibody. The CV method monitored changes in the current signal of the biosensor. The detection linear range was 2.0–240 U mL^−1^, and the detection limit was 0.64 U mL^−1^. 

In addition, Vasudev et al. [68] developed a label-free electrochemical biosensor for the detection of epidermal growth factor receptor (EGFR). The self-assemble monolayer (SAM) of dithiobissuccinimidyl propionate (DTSP) was modified on the surface of the gold electrode. By binding the NH_2_ group of the DTSP-SAM with the carboxylic group of the EGFR antibody, the antibody was immobilized on the electrode to achieve specific capture of the EGFR and the charge transfer onto the modified electrode was enhanced. The changes in the electrical signal during detection of EGFR were monitored by the CV method. The linear range was 1 pg mL^−1^–100 ng mL^−1^. The detection limit of the biosensor was 1 pg mL^−1^.

**Figure 2:**
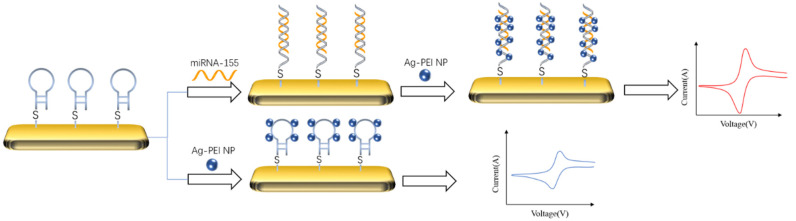
Schematic diagram of detecting miRNA-155 by cyclic voltammetry and signal amplification with Ag-PEI-NP [69]. The nanoparticles could bind to the target captured by the probe to amplify its CV signal.

The typical idea of an electrochemical biosensor for detecting DNA is to capture the target by DNA probe, amplify the signal with the material that can bind with double-stranded DNA. As shown in Figure 2, Hakimian et al. [69] modified the thiolated DNA probe to the gold electrode surface by a gold sulfur bond. Then, the detection target miRNA-155 would specifically bind with the probe to form the double-stranded body. Polyvinylimine silver nanoparticles (Ag-PEI NP) were added to specifically bind with the double-stranded body to amplify the peak current of the biosensor. The detection range of the biosensor was 2 × 10^−20^–2 × 10^−12^ M, and the detection limit was 2 × 10^−20^ M.

#### 3.1.2. Differential Pulse Voltammetry

Differential pulse voltammetry (DPV) is a voltametric technique that combines the potential step technique with the linear potential sweep technique to detect trace target compounds with high sensitivity. The peak height of the DPV curve is related to the concentration of the analyte [7,70].

**Figure 3:**
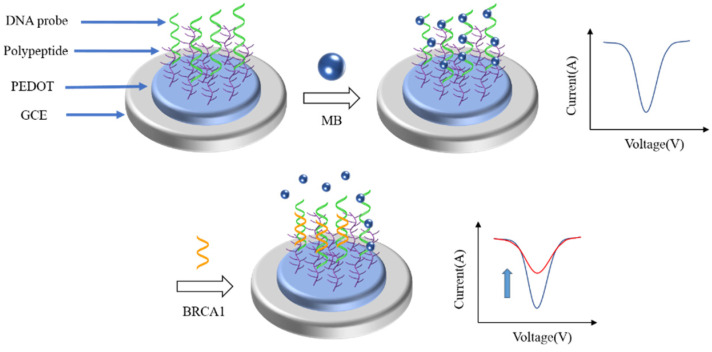
Schematic illustration of the fabrication process of the antifouling BRCA1 biosensor based on PEDOT/PEP and signal amplification with MB [70]. When the target binds to the probe, the MB would fall off the probe, resulting in a significant change in the DPV signal of the biosensor.

At present, various DPV biosensors have been reported to detect different breast tumor markers. As shown in Figure 3, Wang et al. [70] reported a low fouling DPV biosensor for the detection of BRCA1. They modified the DNA probe onto the electrode surface by polypeptide doped poly(3,4-ethylenedioxythiophene) (PEDOT) to capture the BRCA1. Before the introduction of BRCA1, methylene blue was added to connect with the DNA probe to enlarge the DPV signal. When BRCA1 was captured by the DNA probe, methylene blue (MB) would be released from the DNA probe, there would be a large change in the DPV signal. The degree of change in the DPV signal was directly correlated with the concentration of BRCA1. The linear range of the biosensor was 0.01 pM–1 nM. The detection limit was 0.0034 pM.

Han et al. [71] similarly constructed an electrochemical biosensor for the detection of CA15-3 using PEDOT and peptides. PEDOT doped with peptides was modified on the electrode surface to give the biosensor high sensitivity and long-term stability. The biosensor was able to detect CA15-3 in serum without suffering from biofouling. The linear range was 0.01–1000 U mL^−1^. The detection limit was 3.34 mU mL^−1^.

Similarly, another DPV biosensor was reported to detect BRCA1. Xia et al. [72] modified 3D reduced graphene oxide and polyaniline nanocomposites (3D-rGO-PANI) on the surface of a glassy carbon electrode as a sensing layer to improve the conductivity and electrochemical activity of the biosensor. After the target DNA BRCA1 was captured by the DNA probe that was modified on the surface of the 3D-rGO-PANI, the methylene blue would be used to further amplify the DPV signal of the biosensor. The linear range of detection was 1.0 × 10^−15^–1.0 × 10^−7^ M and the detection limit was 3.01 × 10^−16^ M.

For detecting different targets simultaneously, Chang et al. [73] synthesized two different metal–organic frameworks (MOFs) to detect two different RNA. MOFs were packed with different electrochemical dyes with different peak currents. The surface of the MOFs was modified with two different RNA (let-7a and miRNA-21) probes. Specific targets would stimulate specific MOFs to release organic molecules to produce specific peak currents. The linear ranges of detection were 0.01–10 pM (let-7a) and 0.02–10 pM (miRNA-21), respectively. The detection limit was 3.6 fM (let-7a) and 8.2 fM (miRNA-21), respectively.

#### 3.1.3. Square Wave Voltammetry

The waveform of square wave voltammetry can be regarded as a special case of DPV, which is an asymmetrical ladder type. The duration of the pre-electrolysis and pulse cycle is equal. At the end of each pulse, the current is sampled twice in each cycle [74].

Various sensitive biosensor-based on SWV techniques were reported for detecting breast tumor markers. Different from the common detection strategy, Wang et al. [75] used competitive recognition to let the target trigger competitive recognition, which made the previously bound molecules fall off, resulting in a change in the current detected by the biosensor. The DNA probe that was modified with ferrocene at one end was modified on the surface of MXene (Ti_3_C_2_) to connect with the MUC1 aptamer that was modified on the surface of the glassy carbon electrode. After the addition of MUC1, competitive recognition was triggered to change the conformation of the aptamer. The complementary DNA probe was separated from the aptamer, resulting in the combination of MUC1 and the aptamer. The current change in this process was detected by SWV. The linear range of the biosensor was 1.0 pM–10 μM, and the detection limit was 0.33 pM.

To detect multiple microRNAs at the same time, Xu et al. [76] designed a circular DNA probe that can simultaneously detect miRNA-21 and miRNA-155. The probe was fixed on the top of the DNA tetrahedron that was modified on the surface of the electrode to simultaneously recognize miRNA-21 and miRNA-155 through multiple target recognition domains under the assistance of Helper strands, which could trigger mimetic proximity ligation assay (mPLA) for capturing the beacons ferrocene (Fc)-A1 and methylene blue (MB)-A2 to achieve multiple miRNAs detection. The linear range of the biosensor detection was 0.1 fM–10 nM. The detection limits were 18.9 aM (miRNA-21) and 39.6 aM (miRNA-155), respectively.

#### 3.1.4. Linear Sweep Voltammetry

Linear sweep voltammetry is to apply a linearly varying voltage on the electrode and record the current at different electrode potentials. When the electrode potential reaches the potential of the redox reaction, a current peak will be generated. At other potentials, the current changes slowly. This peak is used to quantify the reactant concentration.

For example, Marques et al. [77] developed a biosensor for the detection of the HER2 extracellular domain (ECD) in human serum based on the gold nanoparticle-modified screen-printed carbon electrode. HER2-ECD antibodies were modified on the electrode surface to capture the target. After the target had been captured, the antibody modified with biotin was added to bind with the target to form a sandwich structure. Then, a streptavidin–alkaline phosphatase conjugate was used to connect with the biotin to label the detection antibody. Finally, the enzyme substrate and silver ions were added to enhance the signal. The detection of the target was achieved by linear scanning voltammetry, which detects the electrical signal of the reduction of silver ions to metallic silver during the enzymatic reaction. The linear range was 15 to 100 ng mL^−1^. The detection limit was 4.4 ng mL^−1^.

Due to the direct signal being weak, Freitas et al. [78] designed an LSV biosensor to detect the indirect signal. The screen-printed electrode was modified with multi-walled carbon nanotubes (MWCN), AuNPs, and HER2 antibodies, step by step. The alkaline phosphatase was introduced into the electrode surface by a secondary antibody and biotin-streptavidin. The 3-indoloxyphosphate and Ag^+^ were added as substrates for the enzymatic reaction. The electrochemical signals of silver produced by enzymatic reaction were recorded by LSV as the signal of the biosensor. The signal was directly related to the concentration of HER2. The total assay time was 2 h and 20 min. The linear calibration plots were obtained between 7.5 and 50 ng mL^−1^. The detection limit was 0.16 ng mL^−1^.

In order to improve the detection performance of a sensor, a variety of methods are sometimes used to amplify the signal. Zhao et al. [79] proposed self-assembled supramolecular nanocomposites assisted by a multiple signal amplification strategy to recognize the breast cancer stem cell by the CD44 protein on the cell surface. Firstly, thiol-functionalized peptides were immobilized onto the Au electrode surface to selectively capture the CD44 protein on the cell surface. Lysis of cells occurred after they had been captured, leaving only the captured peptide. Then, trypsin was introduced for the decomposition of peptides that did not capture CD44 and the captured CD44. Then the cucurbit [8] urils were added to bind with the peptides and subsequent addition of AuNPs. Finally, silver deposition was carried out to achieve the final signal amplification. It could also be used to detect the CD44 protein. The detection limit toward CD44 protein was 2.17 pg mL^−1^. The linear range toward CD44 protein was 0.01 ng mL^−1^–100 ng mL^−1^. The linear range toward CD44-positive cells was 10 cells mL^−1^–10^6^ cells mL^−1^. The detection limit toward CD44-positive cells was 8 cells mL^−1^.

#### 3.1.5. Electrochemical Impedance Spectroscopy

The principle of electrochemical impedance spectroscopy is to apply a small amplitude alternating current potential wave with different frequencies to the electrochemical system and measure the ratio of alternating current potential to the current signal, which is the system impedance.

For the EIS biosensor, the detection was based on the impedance change of the electrode. So, the main idea to improve the detection limit was increasing the conductivity or impedance. For instance, Gu et al. [80] fabricated ZrHf-MOFs to amplify the impedance change. The ZrHf-MOFs were coupled with carbon dots to improve the electrochemical activity. The surface of the electrodes was modified with ZrHf-MOFs and aptamers, step by step. Aptamers were modified on the carbon dots to specifically capture the HER2 and HER2-overexpressed living cancer cells MCF-7. The linear range of HER2 detection was 0.001–10 ng mL^−1^. The detection limit was 19 fg mL^−1^. The linear range of MCF-7 cell detection was 1 × 10^2^–1 × 10^5^ cells mL^−1^ and the detection limit was 23 cells mL^−1^.

To amplify the signal, Paimard et al. [81] modified the electrode surface with nanofibers, MWCN, and AuNPs. Nanofibers are inherently large surface area and could increase conductivity and high porosity. MWCN improved the electron transfer rate and catalytic rate in the electrochemical reaction. AuNPs were used to modify the aptamer to capture MUC1 and perform signal amplification. The linear range of the biosensor was 5–115 nM. The detection limit was 2.7 nM.

Shahrokhian et al. [82] combined redox graphene and a conductive polymer to make a DNA biosensor for the detection of BRCA1. The surface of the redox graphene covered with a conductive polymer had more reactive sites for immobilization of the DNA probe, increasing the detection limit of the sensor. The linear range was 10 fM–0.1 µM, and the detection limit was 3 fM.

#### 3.1.6. Field-Effect Sensor

The principle of the FET biosensor is that the gate of the FET is replaced by an ion-selective membrane, reference electrode, and electrolyte. When a fixed voltage is applied to the reference electrode, different ion concentrations and different selective films in the electrolyte result in different interface potentials at the gate insulating layer (equivalent to applying a gate voltage to the gate), which further leads to the changes in carrier distribution in the channel and source leakage current, and the change of current is proportional to the amount of target [83,84]. 

**Figure 4:**
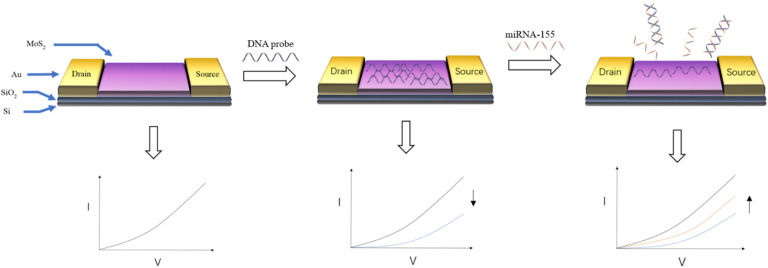
Schematic diagram of detecting miRNA-155 by MoS_2_ FET [85]. Hybridization of the target DNA and the DNA probe causes the DNA probe to be detached from the MoS_2_, causing a change in the target signal.

For FET biosensors, choosing different materials as conductive channels will directly affect the construction of biosensors and the design of detection strategies. At present, many nanomaterials have been selected as conductive channels for FET due to their excellent conductivity and biocompatibility. As shown in Figure 4, Majd et al. [85] used molybdenum disulfide (MoS_2_) as the conductive channel of FET. The DNA probe of miRNA-155 was fixed on the surface of MoS_2_ by physical adsorption. After the probe and DNA combine to form double-stranded DNA, it would fall off from molybdenum disulfide, resulting in a change in the biosensor signal. The detection limit of miRNA-155 was 0.03 fM, and the linear range of detection was 0.1 fM–10 nM. Bao et al. [86] used silicon nanowires as the conductive channel of FET. APTES with glutamic acid (Glu) is used as the connector to connect silicon nanowires and CEA antibodies. The current of FET decreased when the CEA antibody captured the CEA. The decrease part was used to calculate the concentration of CEA. The linear range of detection was 0.1–100 ng mL^−1^, and the detection limit was 10 pg mL^−1^.

The specific detection of the target can be realized through the modification of specific receptors onto the sensing layer. By this method, the influence of other substances in the sample can be reduced and the sample can be detected directly without pretreatment, but the lower detection limit of the sensor will be somewhat increased. Secondary antibodies and nanomaterials can be used to amplify the change in the electrical signal of the biosensor. When the direct signal is weak, target detection can also be achieved by detecting the indirect signal.

### 3.2. Optical Biosensor

The optical biosensor realizes quantitative detection of targets by detecting the optical change on the surface of the sensing layer. Different optical biosensors detect the different optical signals, such as refractive index, resonance, wavelength, intensity, etc. [15,87,88]. In recent years, a number of optical biosensors have been reported for the detection of breast cancer tumor markers, which are classified into fluorescent biosensors, colorimetric biosensors, surface plasmon resonance biosensors, surface Raman scattering sensors, and electrochemiluminescent biosensors, based on the detection method [14,15,87,88].

#### 3.2.1. Fluorescence Biosensor

The principle of the fluorescent biosensor is based on the fluorescence characteristics of molecules, such as the inherent fluorescence characteristics of many proteins and other biological molecules (nucleic acids, NADH, flavin nucleotides, green fluorescent protein). Once the ligands are combined with these proteins, the fluorescence behavior of the molecules will change, thereby realizing the detection of the target.

In contrast, most analytes are nonfluorescent. Therefore, it is necessary to use different fluorescent labels or probes to perform fluorescence spectroscopy detection [9]. The key to using fluorescence to detect the target lies in the change in fluorescence behavior before and after capturing the target, such as fluorescence excitation, quenching, or fluorescence intensity change. 

**Figure 5:**
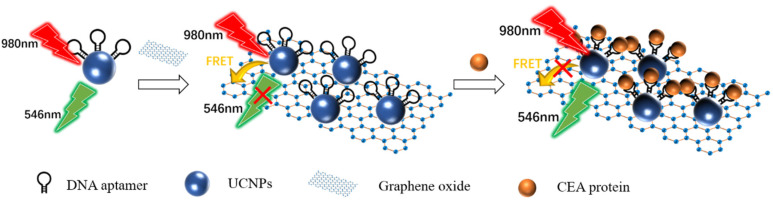
Schematic diagram of detecting the CEA protein using fluorescence biosensor-based UCNPs and FRET [89]. The presence of the target blocked the FRET between the UCNPs and the GO, resulting in the recovery of fluorescence at 546 nm.

As shown in Figure 5, Wang et al. [89] designed an ultra-sensitive homogeneous aptamer biosensor for CEA detection based on the fluorescence resonance energy transfer (FRET) between the upconversion nanoparticles (UCNPs) and graphene oxide (GO). UCNPs modified with a CEA aptamer were immobilized on the GO surface by π–π stacking. Due to the FRET between UCNPs and GO, the 546 nm fluorescence of UCNPs that was excited by the 980 nm laser was quenched. The binding of CEA to the aptamer blocked the π–π stacking. The UCNPs separated from GO. The 546 nm fluorescence recovered. The degree of fluorescence recovery was related to the concentration of CEA. The linear range of the biosensor was 0.03–6 ng mL^−1^. The detection limit was 7.9 pg mL^−1^. The linear range of detection in human serum samples was 0.03–6 ng mL^−1^, and the detection limit was 10.7 pg mL^−1^. 

Not only the fluorescence excitation and quenching, but the intensities of fluorescence could also be used to detect the target. For instance, Mohammadi et al. [90] fabricated carbon dots–chitosan nanocomposite hydrogels which functionalized with the ssDNA probe to detect microRNA-21 in MCF-7 cancer cells. After miRNA-21 were captured, the intensity of the excited fluorescence would decrease. There was a proportional relationship between the change degree in fluorescence intensity and the logarithm of the target concentration. The detection linear range of the biosensor was 0.1–125 fM, with a detection limit of 0.03 fM.

#### 3.2.2. Colorimetric Biosensor

The colorimetric biosensor is based on the characteristic that there is a functional relationship between the color and concentration of the colored substance solution. After the target with different concentrations is captured, it will produce different degrees of color changes. Then the detection of the target concentration is realized.

However, as a result of the small concentration of the target, the direct signal of the colorimetric biosensor is not obvious enough. There were many ways that had been reported, such as detecting the indirect signal and amplifying the direct signal. Bai et al. [91] developed a colorimetric biosensor that detected the indirect signal. The biosensor was divided into two parts: a signal unit and capture unit. The signal unit was responsible for providing detection signals and the capture unit was used to capture the target DNA BRCA1. The capture probe was modified on the surface of the gold-plated silicon wafer to form the capture unit. The signal unit was a two-dimensional nanomaterial Bi_2_Se_3_ nanosheet with AuNPs modified on the surface. The surface of the AuNPs was modified with a signal probe to combine with the BRCA1 that had been captured by the capture probe. The capture unit, signal unit, and target DNA form a sandwich construction. The signal unit could catalyze the reduction in the colorimetric substrate 4-nitrophenol (4-NP). The dynamic constant of the reaction had a good linear relationship with the concentration of BRCA1 between 10^−12^ and 10^−18^ M, and the detection limit was 10^−18^ M.

There were various methods that could be used to amplify the signal of the colorimetric biosensor, such as metal-enhanced fluorescence. Choi et al. [92] developed a CRISPR–CAS12a-based nucleic acid amplification-free fluorescent biosensor to detect cfDNA via AuNPs-assisted MEF and colorimetric analysis. A special double-stranded DNA with a length of 7 nm was prepared to connect AuNPs with a diameter of 20 nm and AuNPs with a diameter of 60 nm. One end of the double-stranded DNA was directly connected to AuNPs with a diameter of 20 nm. The other end was modified with a single-stranded DNA with a length of 2 nm and fluorescein isothiocyanate (FITC). The single-stranded DNA was used to connect AuNPs with a diameter of 60 nm. FITC was used for the fluorescent indicator. Because FITC was too close to AuNPs with a diameter of 60 nm, FITC fluorescence quenching occurs. In the absence of BRCA1, CRISPR–CAS12a had no effect on the fluorescence behavior of FITC. When BRCA1 appeared, CRISPR–CAS12a would combine with BRCA1 to form a CRISPR–CAS12a complex to degrade the single-stranded DNA. The FITC would be far away from the 60 nm AuNPs. Then, the fluorescence of FITC would recover and the color of the solution changed from purple to red-purple. Without any DNA amplification, the biosensor could complete the detection within 30 min with a detection limit of 0.34 fM. The linear range was 1 fM–100 pM.

#### 3.2.3. Surface Plasmon Resonance Imaging

A surface plasmon resonance imaging (SPRi) biosensor is based on the change in the refractive index near the metal–dielectric interface caused by the specific reaction of biomolecules at the metal–dielectric interface. The change in the propagation constant of surface plasmon would lead to the change in the coupling condition between the light wave and surface plasmon. The final measurement object is the change in the characteristic of the light wave interacting with the surface plasma [93].

**Figure 6:**
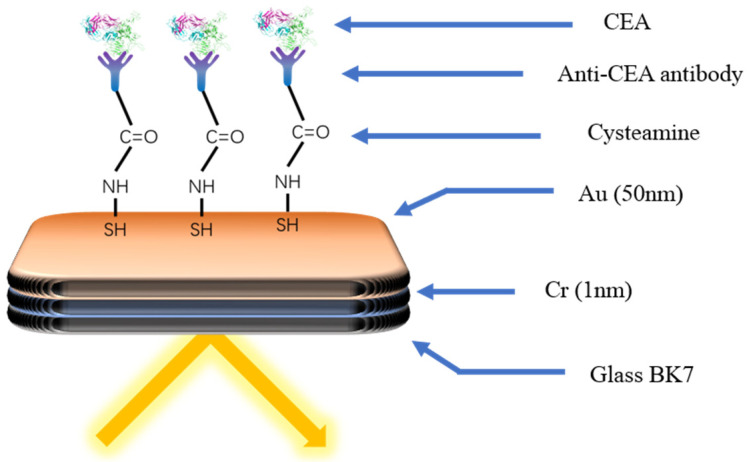
Schematic diagram of the immune biosensor for CEA detection [94]. The specific antibody was modified onto the gold surface through cysteamine. Capture of the target by the antibody causes a change in the light signal.

The typical idea of the SPRi biosensor is modifying the surface of the chip with the specific receptor to capture the target directly, which would bring about the change in the signal, such as the intensities of the light. As shown in Figure 6, Szymanska et al. [94] developed a SPRi biosensor to detect the CEA. The cysteamine was modified on the surface of the gold chip as the connector. The EDC/NHS was used to modify the other end of cysteamine with the antibody to capture the CEA specifically. The linear range of the biosensor was 0.40–20 ng mL^−1^. The detection limit was 0.12 ng mL^−1^. The required sample size was only 3 µL.

Besides proteins, SPRi can also detect other targets, such as exosomes. Sina et al. [95] used biotin-streptavidin to modify the HER2 antibody on the chip surface to detect HER2-positive exosomes. The linear range of the biosensor was 8280 exosomes μL^−1^–33,100 exosomes μL^−1^. The detection limit was 8280 exosomes μL^−1^. In order to improve the detection sensitivity, it is necessary to use signal amplification technology. Moreover, secondary amplification is required while the signal is still small after the first amplification. Wang et al. [96] modified the DNA probe on the surface of the gold electrode to capture exosomes. After the DNA probe captured the exosome, AuNPs were modified with exosome aptamer and the nucleic acid sequence T_30_ that could bind with nucleic acid sequence A_30_. Other AuNPs modified with nucleic acid sequence A_30_ were used for secondary amplification. After the secondary amplification, the detection limit of the exosomes reached 5000 exosomes mL^−1^.

#### 3.2.4. Surface-Enhanced Raman Spectroscopy

Surface-enhanced Raman spectroscopy (SERS) is a kind of surface plasmon resonance effect produced by the analyte and metal plasma adsorbed on the substrate surface under the irradiation of a specific frequency incident laser, which leads to the obvious enhancement of the Raman scattering signal. The reaction between the probe and target would affect the Raman spectra. The change degree of the absorption peak reflects the concentration of the target.

The SERS biosensor is facing the same problem as other biosensors, such as a low primary signal and detecting multiple targets. For amplifying the primary signal of the biosensor, Han et al. [97] prepared a Au/Ag hybrid porous GaN and used it as the substrate of the SERS chip. The DNA probe with one end sulfhydrylated was modified on the chip surface to capture mir-k12-5-5p. With the amplification of the substrate, the detection limit of the biosensor got to 884 pM.

In order to detect multiple microRNAs simultaneously, Wang et al. [98] developed a SERS biosensor based on plasma coupling interference (PCI). PCI depends on the formation of the nano network. The nanoparticles modified with single-stranded capture DNA and Raman-labeled single-stranded receptor DNA, respectively, could form the nano network through the interconnection of DNA. After the formation of the nano network, the PCI effect would cause the Raman label to produce a strong SERS signal in the absence of the target DNA. The target DNA would affect the formation of the nano network through the competitive binding, resulting in the weakening of the SERS signal. By adjusting the DNA sequence used to form the nano network, the detection of different targets can be realized. By using different Raman labels for different detection targets, the biosensor can detect multiple microRNAs simultaneously.

#### 3.2.5. Electrochemiluminescence Biosensor

The principle of electrochemiluminescence is to use the specific luminescence reaction induced by electrochemistry on the surface of the electrode. There is a functional relationship between the luminous intensity and the amount of the target [8].

In order to improve the detection limit of the ECL biosensor, a variety of detection strategies and appropriate signal amplification methods had been reported. Wang et al. [99] developed a super-sensitive biosensor for BRCA1 detection through ZnMOF (Ru) to amplify the ECL signal. The DNA probe with biotin, BRCA1, and ZnMOF (Ru) formed a sandwich construction. The linear range of biosensor detection was 1.0 fM–0.1 nM. The detection limit was 0.71 fM.

Similarly, in the case that the direct signal was too small to detect, the strategy of detecting indirect signals was also used in the ECL biosensor. Qiao et al. [100] developed an ECL biosensor to detect the EXO by the indirect signal. Mercaptopropionic acid-modified Eu^3+^-doped CdS nanocrystals (MPA-CdS: Eu NCs) and H_2_O_2_ were used as the ECL emitters and co-reactant. The surface of the electrode was modified with MPA-CdS: Eu NCs and the CD63 aptamer to capture the EXO secreted by the MCF-7 cells. The second DNA sequence, which had the CD63 aptamer sequence and could fold into G-tetrahedron/heme deoxyribozyme, would capture the EXO that had been captured on the surface of the electrode, and the ECL signal hardly changed. With adding the heme and K^+^, the second DNA sequence would fold into the G-tetrahedron/heme deoxyribozyme to catalyze the decomposition of H_2_O_2_, resulting in a significant reduction in the ECL signal. The degree of ECL signal reduction was linear to the logarithm of the exosome concentration. The linear range of the test is 3.4 × 10^5^–1.7 × 10^8^ exosomes mL^−1^. The detection limit was 7.41 × 10^4^ exosomes mL^−1^.

For improving the detection limit of the ECL biosensor, Cui et al. [101] amplified indirect signals with signal amplification techniques. This ECL biosensor was based on the double signal amplification of an isotherstranded display polymerase reaction (ISDPR) and bridge DNA–AuNPs nanocomposites. The detection strategy was to decompose the target RNA into a large amount of auxiliary DNA by ISDPR reaction and then detect it and amplify the signal. The electrode surface was modified with the DNA probe to capture the auxiliary DNA. The bridge DNA–AuNPs nanocomposites were added to bind to the captured auxiliary DNA for primary signal amplification. Afterward, the streptavidin-modified Ru(dcbpy)_3_^2+^ complex would bind to biotin-labeled single-stranded DNA on AuNPs to achieve secondary amplification. The linear range of biosensor detection was 0.01–10,000 fM. The detection limit was 3.2 aM.

In summary, optical biosensors are also capable of detecting the full class of breast tumor markers. The surface of the chip is functionalized by the receptor to achieve the specific capture of the target. The signal amplification could be realized by carbon dots, graphene quantum dots, AuNPs, MOF, enzymatic reaction, MWCN, nanocrystals, etc. The sensitivity of the optical biosensor is equivalent to the electrochemical biosensor.

### 3.3. Other Types of Biosensors

In addition to electrochemical and optical biosensors, there are also quartz crystal microbalance (QCM) biosensor and photoelectrochemical biosensor. QCM is used to detect the mass change of the target. The photoelectrochemical (PEC) biosensor is used to detect the effect of the targets on the photoelectric characteristics of materials.

#### 3.3.1. QCM Biosensor

The sensing principle of QCM is based on the effect of the target on the frequencies of the bulk acoustic waves generated in the piezoelectric quartz crystal. The frequency change of the acoustic wave is related to the mass change on the chip surface, so as to realize the concentration detection of the target. QCM is able to detect nanogram-level mass changes on the chip surface [102].

**Figure 7:**
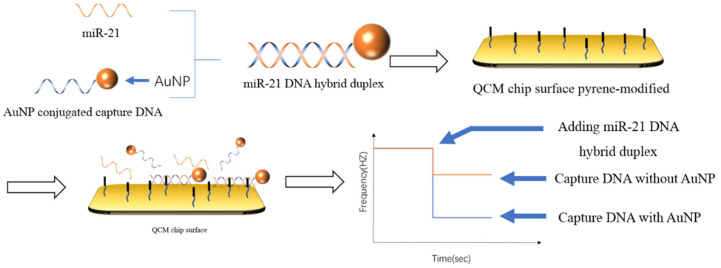
Schematic illustration of the QCM biosensor for miRNA detection [104]. The DNA that had formed a double strand was captured by the DNA embedding agent modified on the surface of the chip, and the AuNPs modified at the end of the probe were used to amplify the mass change on the chip surface.

By modifying different receptors on the surface of QCM chip, QCM can detect different kinds of tumor markers. For instance, Yang et al. [103] modified the polydopamine and polyethyleneimine composite membrane and hyaluronic acid on the QCM chip surface to capture the CD44 protein on the surface of breast cancer cells. The detection limits of MDA-MB-231 (M231) cells and MCF-7 cells were 300 cells mL^−1^ and 1000 cells mL^−1^, respectively. In contrast to capturing the target DNA with a DNA probe, as shown in Figure 7, Park et al. [104] chose to capture the double-stranded body formed by the binding of the target and the probe. Considering the mass of DNA was too small, the DNA probe was modified on the surface of the AuNPs to increase the weight of the double-stranded DNA. The detection time was 105 min. The detection limit was 3.6 pM. The detection linear range was 2.5 pM–2.5 μM.

The use of the second antibody could also amplify the mass change on the QCM chip surface. Lin et al. [105] used a sandwich detection strategy to realize the detection and signal amplification of CA15-3. First, a two-dimensional nanomaterial MoS_2_ film was coated on the surface of the QCM chip, and the first antibody of CA15-3 was modified on the MoS_2_ film by physical adsorption. Then the CA15-3 was added and captured by the first antibody. Subsequently colloidal gold with the second antibody of CA15-3 modified was added to combine with the captured CA15-3 for signal amplification. The linear range of the biosensor was 0.5–100 U mL^−1^, and the detection limit was 0.5 U mL^−1^.

In addition, considering the antibody capture efficiency, it is important to increase the quantity of the antibody that was modified on the surface of the QCM chip. Bakhshpour et al. [106] modified 2-hydroxyethyl methacrylate-PHEMA nanoparticles on the surface of QCM chip to increase the surface area of the chip to increase the quantity of the antibody. Then, the Notch-4 receptor antibody was modified on the chip surface by carbodiimide to capture the MDA MB 231 cancer cells specifically. The detection limit of the biosensor was 12 cells mL^−1^.

#### 3.3.2. Photoelectrochemical Biosensor

The principle of the PEC sensor is that when the light irradiates the photoelectric active material, the electrons in the material are excited, leading to a photocurrent or photovoltage. The recognition probe on the surface of the photoelectric active material captures the target, which would cause the change in the photocurrent or photovoltage. When the concentration of the target changes, the photoelectric signal changes accordingly. There is a relationship between the concentration of the target and the photoelectric signal. Various PEC biosensors had been reported to detect tumor markers.

Guo et al. [107] developed a dual-signal amplification HER2 PEC biosensor. The biosensor was prepared based on the tungsten sulfide nanowire array on Ti mesh (WS_2_ NW/TM). The HER2 aptamer was wrapped onto the WS_2_ NW/TM surface for specific capture of the HER2 molecules. The signal amplification was achieved by the AuNPs that were modified with glucose oxidase (GOx) and HER2-binding peptides. The AuNPs would directly amplify the photocurrent. The GOx catalyzed the decomposition of glucose into hydrogen peroxide would increase the photocurrent. The linear range of the biosensor was 0.5–10 ng mL^−1^. The detection limit was 0.36 ng mL^−1^.

In addition, Fu et al. [108] developed a PEC detection platform for vascular endothelial growth factor 165 (VEGF165) based on the characteristic of the porous Cu_2_O-CuO flowers that could cause photocurrent polarity conversion of CdS quantum dots modified ITO electrode. CdS quantum dots were modified on the surface of the ITO electrode to generate a large anodic current. Then, the hairpin DNA1 (HP1) was modified to capture the DNA S1. The DNA S1 bound with the VEGF165 aptamer. When VEGF165 was captured, the DNA S1 would be released and captured by the HP1. Then, biotin-labeled hairpin DNA2 (bio-hp2) was added to catalyze the hairpin assembly process. A large amount of HP1/bio-hp2 double-stranded DNA would be formed on the electrode surface. The peak value of the photocurrent was linear with the concentration of VEGF165 in the range of 1–3000 fM. The detection limit was 0.3 fM.

In conclusion, QCM and PEC are also capable of detecting all types of tumor markers. The signal amplification of the QCM biosensor is mainly to amplify the mass change of chip surface. The signal amplification of the PEC biosensor is to amplify the change of photocurrent and photovoltage caused by the target. While it is possible to improve the detection limits of QCM and PEC sensors for detecting a single target through signal amplification techniques, they have difficulty detecting multiple targets simultaneously.

## 4. New Strategies for Biosensor

In general, the challenges that biosensors faced mainly come from two aspects: detection strategy and detection device. At the level of detection strategies, biomolecules are generally difficult to cope with harsh environments. For example, the activity and shelf life of biomolecules are often influenced by the environment. At the device level, most biosensors are difficult to detect multiple targets simultaneously and are not sufficiently integrated and miniaturized. The combination of molecularly imprinted polymers (MIPs) and microfluidic chips with biosensing has great potential to address these challenges.

### 4.1. Molecularly Imprinted Polymers

MIPs are polymers processed using molecular imprinting techniques that leave cavities in the polymer matrix and have an affinity for selected ‘template’ molecules. When added to the target, the target binds specifically to the cavity, enabling detection of the target [109]. The greatest advantage of MIPs over biomolecules is their ability to maintain functional stability in relatively harsh environments [110], and the use of MIPs enables the capture of a wide range of targets, from small molecules to large molecular entities, such as pathogens and whole cells [111,112,113]. Therefore, the demand for MIPs in the field of biosensors development is increasing recently and there is a significant improvement.

MIPs have been studied in combination with biosensors to detect breast cancer tumor markers. Ribeiro et al. [114] used molecular imprinting techniques and electrochemical methods to prepare a protein imprinted poly(Toluidine Blue) film with CA15-3 as a template in a pre-formed Toluidine Blue (TB) tailed SAM at the AuSPE surface for the specific detection of CA15-3. Monitoring of current magnitude by the DPV method revealed that the current decreased as the concentration of the target increased. The linear range of detection was 0.10 U mL^−1^–100 U mL^−1^, and the detection limit was below 0.10 U mL^−1^. 

Pacheco et al. [115] used a solution containing phenol and the extracellular domain of the human epidermal growth factor receptor 2 (HER2-ECD) to electropolymerize molecularly imprinted polymers using HER2-ECD as a template by cyclic voltammetry on the surface of screen-printed electrodes. The peak current intensity of the sensor was detected by the DPV and increased with increasing HER2-ECD concentration. The linear range was 10 to 70 ng mL^−1^. The detection limit was 1.6 ng L^−1^ and the limit of quantification was 5.2 ng mL^−1^. In addition, Santos et al. [116] prepared a molecularly imprinted polymer using CA15-3 as a template by electropolymerizing pyrrol around CA15-3 on a fluorine doped tin oxide (FTO) conductive glass support to detect CA15-3. The detection of the concentration of the target was accomplished by potentiometric titration. The linear range was 1.44–13.2 U mL^−1^. The detection limit was 1.07 U mL^−1^.

You et al. [113] designed a new strategy to detect the BRCA1. The surface of the glassy carbon electrode is first covered with a layer of gold nanoparticles-reduced graphene oxide (AuNPs-GO). Then the electrode was covered by the layer of molecularly imprinted polymers (MIPs) synthesized with rhodamine B (RhB) as template, methacrylic acid (MAA) as the monomer, and Nafion as additive. The surface of the SiO_2_ nanoparticles was covered by the silver nanoparticles (AgNPs) in situ (SiO_2_@AgNPs) as the signal amplification tracing tag. By the Ag-S bond, the DNA probe could be modified on the surface of SiO_2_@AgNPs. After the DNA probe bound with BRCA1, the RhB labeled DNA would be introduced to connect with the BRCA1 that had been captured by the DNA probe. Then, the RhB would be specifically recognized by MIPs via the interaction between imprinting cavities and RhB. The linear range was 10 fM–100 nM. The detection limit was 2.53 fM. 

### 4.2. Microfluidic Chip

The microfluidic chip is a microanalysis and detection platform integrating sampling, dilution, reagent addition, reaction, separation, and detection into one chip. In microfluidic devices, microchannels are fabricated on a chip and modified by antibodies to capture the target. An appropriate amount of sample is placed in the sample area of the chip. Due to capillary action, the sample flows through the channel and is separated. The assay is then completed in the detection zone. Pre-treatment, mixing, separation and detection of samples can all be done in a single chip. By constructing multiple channels and modifying each channel with a different antibody, it is possible to detect multiple targets simultaneously. The combination of microfluidics technology and biosensing enables biosensors to detect multiple markers simultaneously [5,117,118].

The researchers combined microfluidic chip with biosensing technology to detect various tumor markers. Gao et al. [119] combined giant magnetoresistance with a microfluidic chip to develop a system that could simultaneously detect 12 tumor markers (AFP, CEA, CYFRA21-1, NSE, SCC, PG I, PG II, CA19-9, total PSA, free PSA, free-β-hCG, and Tg) within 15 min. The linear detection range of the biosensor for different targets was 0.5–500 ng mL^−1^ (CEA), 1–1000 ng mL^−1^ (AFP), 0.1–100 ng mL^−1^ (total PSA), 0.1–50 ng mL^−1^ (free PSA), 2–200 ng mL^−1^ (PG I), 1–100 ng mL^−1^ (PG II), 0.5–100 ng mL^−1^ (CYFRA21-1), 1–200 ng mL^−1^ (NSE), 0.5–200 ng mL^−1^ (free-β-hCG), 0.5–70 ng mL^−1^ (SCC), 5–2000 ng mL^−1^ (Tg) and 4–800 U mL^−1^ (CA19-9) respectively. Zheng et al. [120] had combined microfluidic chips with SERS to detect CA125, CA153, and CEA in human serum samples simultaneously. They prepared three microfluidic channels that were modified with three different antibodies to capture the target. The detection limits of these biosensors were 0.01 U mL^−1^ (CA153), 0.01 U mL^−1^ (CA125) and 1 pg mL^−1^ (CEA), respectively.

## 5. Summary and Prospect

Tumor markers run through the entire process of breast cancer diagnosis and treatment, from early diagnosis, treatment options, disease progression, cancer stage, drug resistance, prognosis evaluation, and so on. However, there is still no tumor marker that could effectively predict breast cancer before clinical symptoms. The high sensitivity and specificity of tumor markers still need further study. The development of biosensors also faces opportunities and challenges. In terms of the targets detected, the biosensor is capable of detecting the full class of tumor markers rather than being limited to breast cancer tumor markers. This is because the essence of different cancer markers are proteins, DNAs, cells, and exosomes. As shown in Table 1, most biosensors can only detect a single target and lack the ability to detect different types of targets simultaneously. It will be a great challenge for the development of biosensors in the future to detect and distinguish multiple targets at the same time.

For the detection specificity and anti-interference ability, the biosensor can ensure strong specificity and high sensitivity in the laboratory, relying on antibodies, DNA probes, aptamers, and so on. However, for the human serum samples, the sensitivity of the biosensor decreased on different levels. This indicate that the substance in the human serum sample has an obvious influence on the sensitivity of the biosensor. If the sample is a human whole blood sample, the sensitivity of the biosensor would decline more significantly. Therefore, improving the detection specificity and anti-interference ability of the biosensor in detecting whole blood samples is also a major requirement for the future biosensor.

For the biosensor sensitivity, the direct signal of the biosensor is usually too small to detect due to three major causes: (1) the low concentration of tumor markers in the sample; (2) the interference of other substances in the sample; and (3) the weak electrochemical signal of the tumor markers. To deal with these problems, three methods have been used. First, novel nanomaterials are used as substrates for biosensors to amplify the raw signal of the biosensor. Second, secondary antibodies and secondary antibody-like substances can be used to amplify the initial signal of the biosensor. Third, detecting the larger indirect signal can be used to cope with these problems. 

From the viewpoint of biosensor operability, the complexity of the biosensor operation should be reduced as much as possible on the basis of ensuring the detection performance of the biosensor. The operation of typical biosensors is relatively simple. While the operability of some biosensors is significantly reduced because they adopt complex detection strategies and signal amplification methods to increase the detection limit. 

In summary, the main challenges faced by biosensors are to improve the sensitivity, enhance the specificity, multi-target detection simultaneously, and simplify the operation. These challenges can be divided into two parts: detection strategy and detection equipment. The detection strategy includes improving the sensitivity and enhancing the specificity. The detection equipment includes multi-target detection simultaneously and simplified operation.

The detection strategy part could be overcome by using new nanomaterials as the substrate material to improve the primary signal [121]. The second antibody could be used to amplify the signal of the biosensor. The strategy of detecting the indirect signal could also be used to improve the sensitivity. Sample pretreatment can solve the problem of background interference to improve the performance of the biosensor. The usage of the MIPs could address the problem that biomolecules have difficulty coping with harsh environments.

The problem of the detection equipment part could be solved by combining the microfluidic chip with the biosensing. The detection principle of microfluidic chip-based biosensor is still the principle of electrochemical and optical biosensors. Most of the microfluidic chip-based biosensors are innovative in devices, while electrochemical and optical biosensors are more innovative in terms of detection strategies and materials. Without considering the processing difficulty and cost, the microfluidic chip-based biosensor could realize multi-target detection and operation simplification. Therefore, the detection principles of electrochemical and optical biosensors can be combined with microfluidic chips to obtain biosensors with better overall performance. At last, microfluidic chip-based biosensors are expected to be commercialized on a large scale in the future and change the current detection paradigm.

## Figures and Tables

**Table 1:** A summary of developments of biosensors for breast cancer tumor markers.

Type of Biosensor			Target	Detection Limit	Linear Range	References
Electrochemical biosensor		CV	CA15-3	0.64 U mL^−1^	2.0–240 U mL^−1^	[67]
			EGFR	1 pg mL^−1^	1 pg mL^−1^–100 ng mL^−1^	[68]
			miRNA-155	2 × 10^−20^ M	2 × 10^−20^–2 × 10^−12^ M	[69]
		DPV	BRCA1	0.0034 pM	0.01 pM–1 nM	[70]
			CA15-3	3.34 mU mL^−1^	0.01–1000 U mL^−1^	[71]
			BRCA1	3.01 × 10^−16^ M	1.0 × 10^−15^–1.0 × 10^−7^ M	[72]
			let-7a miRNA-21	3.6 fM (let-7a) 8.2 fM (miRNA-21)	0.01–10 pM (let-7a) 0.02–10 pM (miRNA-21)	[73]
		SWV	MUC1	0.33 pM	1.0 pM–10 µM	[75]
			miRNA-21 miRNA-155	18.9 aM (miRNA-21) 39.6 aM (miRNA-155)	0.1 fM–10 nM	[76]
		LSV	HER2-ECD	4.4 ng mL^−1^	15–100ng mL^−1^	[77]
			HER2	0.16 ng mL^−1^	7.5–50 ng mL^−1^	[78]
			CD44 CD44 positive cell	2.17 pg mL^−1^ 8 cells mL^−1^	0.01 ng mL^−1^–100 ng mL^−1^ 10 cells mL^−1^–10^6^ cells mL^−1^	[79]
		EIS	HER2 MCF-7 cell	19 fg mL^−1^ 23 cells mL^−1^	0.001–10 ng mL^−1^ 1 × 10^2^–1 × 10^5^ cells mL^−1^	[80]
			MUC1	2.7 nM	5–115 nM	[81]
			BRCA1	3 fM	10 fM–0.1 µM	[82]
		FET	miRNA-155	0.03 fM	0.1 fM–10 nM	[85]
			CEA	10 pg mL^−1^	0.1–100 ng mL^−1^	[86]
Optical biosensor		Fluorescence biosensor	CEA	7.9 pg mL^−1^ (Water) 10.7 pg mL^−1^ (Human serum samples)	0.03–6 ng mL^−1^ (Water) 0.03–6 ng mL^−1^ (Human serum samples)	[89]
			miRNA-21	0.03 fM	0.1–125 fM	[90]
		Colorimetric biosensor	BRCA1	10^−18^ M	10^−12^–10^−18^ M	[91]
			BRCA1	0.34 fM	1 fM–100 pM	[92]
		SPRi	CEA	0.12 ng mL^−1^	0.40–20 ng mL^−1^	[94]
			HER2-positive EXO	8280 exosomes μL^−1^	8280–33,100 exosomes μL^−1^	[95]
			EXO	5000 exosomes mL^−1^	/	[96]
		SERS	miR-K12-5-5p	884 pM	/	[97]
			MicroRNA	/	/	[98]
		ECL	BRCA1	0.71 fM	1.0 fM–0.1 nM	[99]
			EXO	7.41 × 10^4^ exosomes	3.4 × 10^5^–1.7 × 10^8^ exosomes mL^−1^	[100]
			miRNA-21	3.2 aM	0.01–10,000 fM	[101]
QCM			miRNA-21	3.6 pM	2.5 pM–2.5 μM	[104]
			MDA-MB-231 cell MCF-7 cell	300 cells mL^−1^ (M231) 1000 cells mL^−1^ (MCF-7)	1 × 10^3^–5.0 × 10^5^ cells mL^−1^ (M231) 5 × 10^3^–4 × 10^5^ cells mL^−1^ (MCF-7)	[103]
			MDA MB 231 cell	12 cells mL^−1^	50–300 cells ml^−1^	[106]
			CA15-3	0.5 U mL^−1^	0.5–100 U mL^−1^	[105]
PEC			HER2	0.36 ng mL^−1^	0.5–10 ng mL^−1^	[107]
			VEGF165	0.3 fM	1–3000 fM	[108]
MIPs	DPV		CA15-3	0.10 U mL^−1^	0.10 U mL^−1^–100 U mL^−1^	[114]
			HER2-ECD	1.6 ng mL^−1^	10–70 ng mL^−1^	[115]
			BRCA1	2.53 fM	10 fM–100 nM	[113]
	Potentiometric Procedures		CA15-3	1.07 U mL^−1^	1.44–13.2 U mL^−1^	[116]
Microfluidic chip	GMR		CEA AFP total PSA free PSA PG I PG II CYFRA21-1 NSE free-β-hCG SCC Tg CA19-9		0.5–500 ng mL^−1^ (CEA) 1–1000 ng mL^−1^ (AFP) 0.1–100ng mL^−1^ (total PSA) 0.1–50 ng mL^−1^ (free PSA) 2–200 ng mL^−1^ (PG I) 1–100 ng mL^−1^ (PG II) 0.5–100 ng mL^−1^ (CYFRA21-1) 1–200 ng mL^−1^ (NSE) 0.5–200 ng mL^−1^ (free-β-hCG) 0.5–70 ng mL^−1^ (SCC) 5–2000 ng mL^−1^ (Tg) 4–800 U mL^−1^ (CA19-9)	[119]
	SERS		CA125 CA153 CEA	0.01 U mL^−1^ (CA153) 0.01 U mL^−1^ (CA125) 1 pg mL^−1^ (CEA)		[120]

## Data Availability

Not applicable.
